# Performance of a Cyanobacteria Whole Cell-Based Fluorescence Biosensor for Heavy Metal and Pesticide Detection

**DOI:** 10.3390/s130506394

**Published:** 2013-05-14

**Authors:** Wong Ling Shing, Lee Yook Heng, Salmijah Surif

**Affiliations:** 1 Faculty of Science, Technology, Engineering and Mathematics, INTI International University, Nilai, Negeri Sembilan 71800, Malaysia; 2 Faculty of Science and Technology/South-East Asia Disaster Prevention Research Institute, University Kebangsaan Malaysia, Bangi, Selangor 43600, Malaysia; E-Mail: leeyookheng@yahoo.co.uk; 3 Faculty of Science and Technology, University Kebangsaan Malaysia, Bangi, Selangor 43600, Malaysia; E-Mail: salmijahsurif@gmail.com

**Keywords:** whole cell biosensor, pHEMA, heavy metals, pesticides

## Abstract

Whole cell biosensors always face the challenge of low stability of biological components and short storage life. This paper reports the effects of poly(2-hydroxyethyl methacrylate) (pHEMA) immobilization on a whole cell fluorescence biosensor for the detection of heavy metals (Cu, Pb, Cd), and pesticides (dichlorophenoxyacetic acid (2,4-D), and chlorpyrifos). The biosensor was produced by entrapping the cyanobacterium *Anabaena torulosa* on a cellulose membrane, followed by applying a layer of pHEMA, and attaching it to a well. The well was then fixed to an optical probe which was connected to a fluorescence spectrophotometer and an electronic reader. The optimization of the biosensor using several factors such as amount of HEMA and drying temperature were undertaken. The detection limits of biosensor without pHEMA for Cu, Cd, Pb, 2,4-D and chlorpyrifos were 1.195, 0.027, 0.0100, 0.025 and 0.025 μg/L respectively. The presence of pHEMA increased the limits of detection to 1.410, 0.250, 0.500, 0.235 and 0.117 μg/L respectively. pHEMA is known to enhance the reproducibility of the biosensor with average relative standard deviation (RSD) of ±1.76% for all the pollutants tested, 48% better than the biosensor without pHEMA (RSD = ±3.73%). In storability test with Cu 5 μg/L, the biosensor with pHEMA performed 11.5% better than the test without pHEMA on day-10 and 5.2% better on day-25. pHEMA is therefore a good candidate to be used in whole cell biosensors as it increases reproducibility and enhances biosensor storability.

## 1. Introduction

Heavy metals and pesticides are amongst the most significant environment pollutants today [1–3]. These pollutants are chemically different and require different methods of detection. Many lab-based techniques for measuring heavy metals and pesticides are based on spectroscopic and chromatographic methods. Unfortunately, these instruments require skillful operators and are time consuming, when rapid screening of environmental samples is required [4–7].

Biosensors can be an alternative tool for the screening of environmental pollutants, as biosensors are highly sensitive, detect rapidly, are portable and suitable for online operations [5,8–11]. Living organisms are increasingly used as the agent to detect the presence of pollutants, and whole cells are seen to have particular advantages for such environmental monitoring tasks. Whole cell biosensors however have generally been limited by their short storage life and the instability of the cells.

Immobilization of biological elements is important to enhance the performance of biosensors, by bringing the biological elements which serve as the reaction site closer to the transducer [8,12,13]. Immobilization of whole cells can be accomplished either by direct entrapment onto a matrix surface [7,14], or through certain immobilizing agents [15,16] such as polysaccharides (e.g., carrageenan and chitosan), proteins (e.g., gelatin and collagen) or synthetic polymers (e.g., polyvinyl alcohol (PVA) and poly 2-hydroxyethyl methacrylate (pHEMA)). The immobilizing agents can be gelled into hydrophilic matrices under mild conditions, thus allowing cell entrapment with minimal loss of cell viability. Gelation of polysaccharides and proteins generally requires slightly elevated temperatures or non-neutral pH conditions. These requirements are less desirable compared to pHEMA, which can form an immobilization matrix for cells under room temperature and neutral pH conditions.

pHEMA forms a knitted structure after the hydrogel is coated onto the supporting material. The gel helps prevent mechanical leakage of cells and avoid bacterial contamination [17]. Capable of retaining up to 70% of water in its structure, the polymer provides a suitable environment for cellular reactions [18]. The ability to coat the gel under room temperature and neutral conditions makes the polymer a good candidate to be used on biological elements which are susceptible to high temperatures and non-neutral conditions [8,19–21].

pHEMA has been successfully used as immobilizing agent in several enzyme and whole cell biosensors. For example, the immobilization of tyrosinase in the phenolic compounds biosensor using potentiostat as the transducer [22], the immobilization of diamine oxidase in the histamine biosensor with oxygen electrode transduction [23], the immobilization of alanine dehydrogenase in the ammonium ion biosensor with the biological signals transduce by potentiostat [24], and in whole cell applications, Tay *et al.* [8] and Wong *et al.* [16] have successfully immobilized the cyanobacterium *A. torulosa* in biosensors for heavy metal detection, with an oxygen electrode used as the transducer. To date, there are only two published articles regarding the use *A. torulosa* in biosensors [8,16]. The use of immobilized *A. torulosa* with fluorescence transduction as a toxicity biosensor as described in this paper is a topic that has never been explored before.

In this paper, we report the effect of pHEMA as the immobilizing agent in a cyanobacteria-based fluorometric biosensor for heavy metal and pesticide detection. A small portion of energy received by the cyanobacteria from sunlight is converted and released as fluorescence [25]. The presence of any substance such as heavy metals and pesticides that inhibits the photosynthetic electron transport pathways will increase the fluorescence emission because the light energy absorbed during excitation could not be utilized in photosynthetic system and has to be released again. The change in fluorescence emission can be measured with a fluorescence spectrophotometer.

The chlorophyll-containing cyanobacterium *A. torulosa* was chosen in this study as cyanobacteria are able to fluoresce naturally under the exposure of certain excitation wavelengths. The organism also forms filamentous colonies that enable direct entrapment of the colonies onto cellulose membranes without leakage. Experiments on the effects of pHEMA were conducted by coating a layer of pHEMA on top of the immobilized cyanobacteria.

## 2. Experimental

### 2.1. Reagents

Bold Basal Medium [23,24] for bacteria culture and pHEMA with mw = 30.000 were obtained from Sigma (Dorset, UK). 1,4-dioxane and O,O-diethyl O-(3,5,6-trichloro-2-pyridyl)phosphorothioate (chlorpyriphos) were purchased from Fisher Scientific (Longhborough, UK). Copper nitrate Cu(NO_3_)_2_, lead nitrate Pb(NO_3_)_2_, and cadmium nitrate Cd(NO_3_)_2_ were purchased from Merck (Darmstadt, Germany), while 2,4-dichlorophenoxyacetic acid (2,4-D) was purchased from Fisher (Pittsburgh, PA, USA). All solutions were prepared in deionized distilled water. All the glassware used was autoclaved at 121 °C for 15 minutes.

### 2.2. Construction of the Biosensor

**Figure 1.**
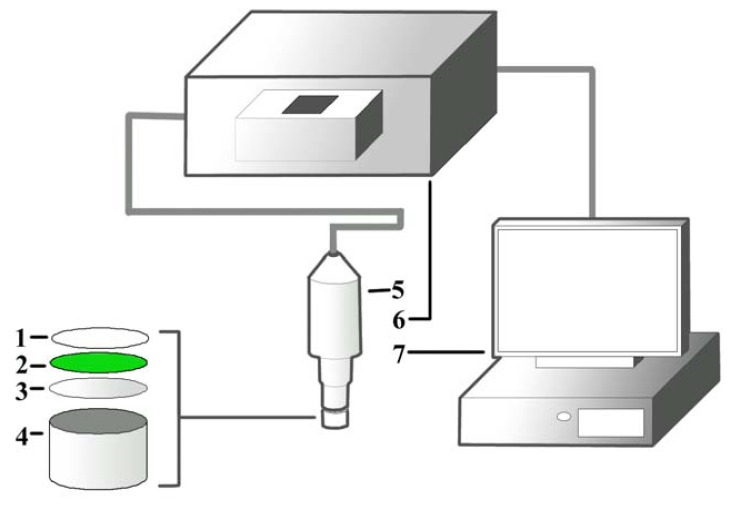
The hydrogel pHEMA (1) immobilizes the cyanobacteria (2) on a cellulose membrane (3) which was then attached to a well with d ≈ 0.8 cm (4). The well was attached to the optical probe (5), which was connected to the fluorescence spectrophotometer (6) and computer (7) for the detection of analytes (heavy metals and pesticides).

The cyanobacateria *A. torulosa* (Carolina Biological Supply Co., Burlington, NC, USA) was cultivated in Bold Basic Medium in a growth chamber (GC500, Protech, Seri Kembangan, Malaysia) at 18.5 °C, with 1,000 Watt/m^2^ white fluorescent illumination and light/darkness maintained at 16/8 hours. The culture was manually agitated for better aeration and to prevent cells from clumping. A suspension containing 5.0 mL of day-7 culture (OD700 nm = 0.3 Abs) was directly entrapped onto a cellulose membrane via filtration with a vacuum pump. The membrane was later air-dried at 18.5 °C and punched into small discs (diameter, d = 0.6 cm). This is followed by adding of 20.0 μL of pHEMA solution (20.0 mg/mL, with H_2_O: 1,4-dioxane = 4:1) on the surface of the membrane and the discs were left to dry for 12 hours at 18.5 °C to immobilize the cells. Individual dried discs were then attached to a well with d ≈ 0.8 cm and fixed onto an optical probe connected to a fluorescence spectrophotometer for measuring the fluorescence signal after excitation. The design of the biosensor containing the pHEMA is illustrated in Figure 1.

### 2.3. Operation of the Biosensor

*A. torulosa* in the well was activated by adding 10 μL of distilled water. For toxicity tests, a volume of 20 μL of Cu, Pb, Cd, 2,4-D, and chlorpyriphos at varying concentrations were added to the well, respectively. The exposure time was set to 30 minutes. Fluorescence intensity was measured before and after the exposure of the toxicants, using a fluorescence spectrometer (LS 55, PerkinElmer, Wiesbaden, Germany), with the excitation and emission wavelengths set to 526 nm and 648 nm, respectively. The biosensor was working at room temperature at pH 7. A volume of 20 μL of distilled water was added to a cell-containing well as control. The percentage of increase in fluorescence after the exposure was determined using the following equation:

%increase of fluorescence=%increase of fluorescence for analyte–%increase of fluorescence for blank

The effects of pHEMA on the detection limits, sensitivity, reproducibility, and the storability of the biosensor were also determined.

## 3. Results and Discussion

### 3.1. Cyanobacteria Culture and Cell Optimization

Bold Basal Medium was modified from the Bristol Medium, which contained all macronutrients and micronutrients necessary for the optimum growth of cyanobacteria and for long term cultivation of algae and cyanobacteria [26,27]. Cyanobacteria from the exponential growth phase were used throughout the experiment. The number of cells was determined according to optical density (OD) determination at 700 nm, which can be linearly correlated to the number of cyanobacteria [8,28]. The method is faster and easier than cell counting by hemocytometer. The same method was also used for cell number determination for algae and bioluminescence bacteria [7,29].

**Figure 2.**
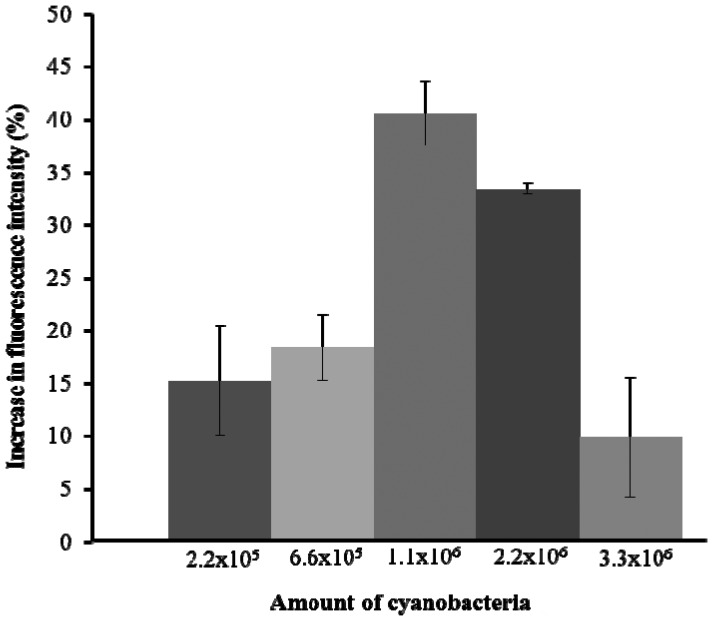
Fluorescence yields from different amounts of immobilized cyanobacteria.

The initial experiment was to test the fluorescence emission capacity of *A. tolurosa*. Five different amounts of *A. torulosa* (2.2 × 10^5^, 6.6 × 10^5^, 1.1 × 10^6^, 2.2 × 10^6^, and 3.3 × 10^6^) were immobilized onto the cellulose membrane. The experiment was carried out in triplicate. Membrane with 1.1 × 10^6^ cells yielded the highest fluorescence emission (Figure 2), with fluorescence intensity decreasing at higher concentrations of cells. This was likely due to reabsorption of the emitted fluorescence by neighboring cyanobacteria [7,30]. Thus the immobilized cells used in all subsequent experiments were set at 1.1 × 10^6^ cells.

### 3.2. pHEMA Optimization

**Figure 3.**
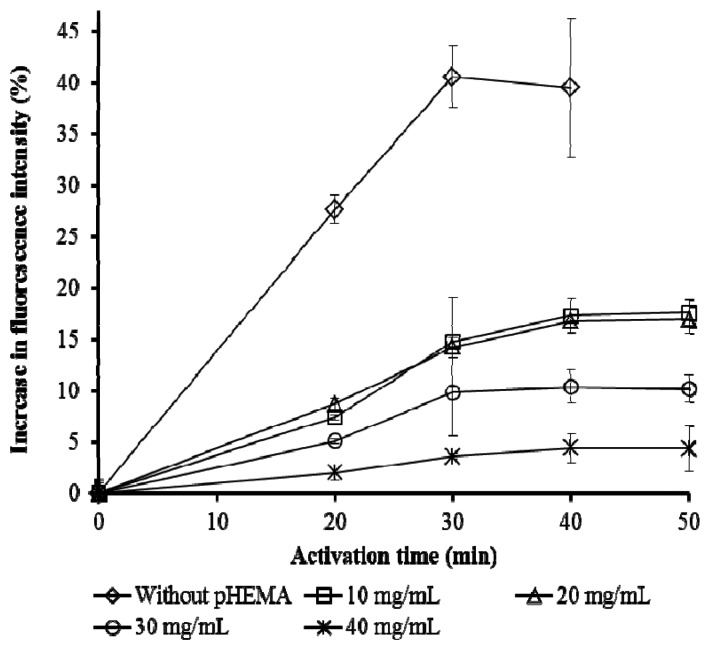
Effect of different amount of pHEMA on activation time (n = 3).

The presence of pHEMA significantly reduced fluorescence yield, at the same time lengthening the activation time (Figure 3). These effects were expected as the presence of an extra layer of hydrogel increases scattering of the emitted fluorescence. The water added for the activation of the cells will need time to permeate through the hydrogel layer to reach the cells. pHEMA exceeding 20 mg/mL reduced the fluorescence significantly. Thus 20 mg/mL pHEMA was taken as the optimized concentration for the immobilization while the activation time was set at 40 minutes. Activation is necessary to improve the performance of whole cell biosensors [7,31].

**Figure 4.**
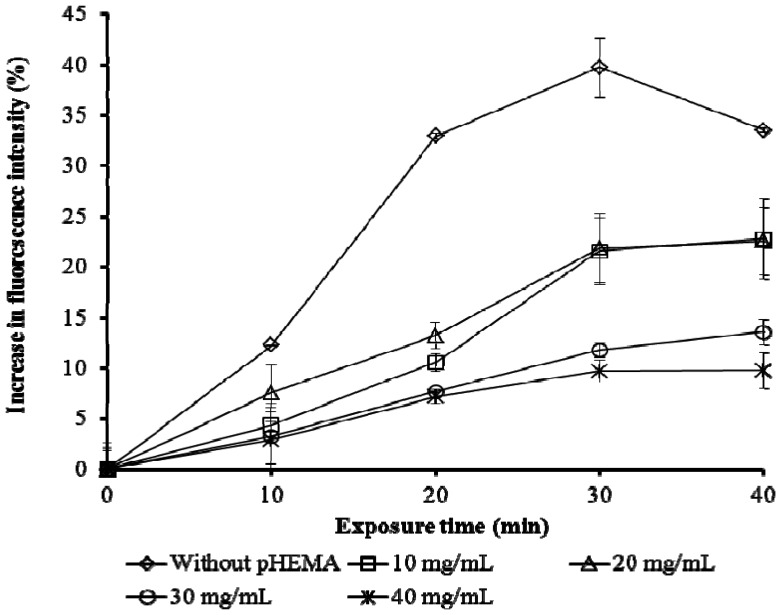
Exposure time of biosensor with different amount of pHEMA, with 10 μg/L of Cu.

Different amounts of pHEMA did not affect the time taken to reach maximum intensity of fluorescence to Cu as shown in Figure 4. In all cases, the highest yield of fluorescence was achieved within 30 minutes. However, without pHEMA, a decrease in fluorescence after 30 min exposure to Cu was observed. Therefore 30 minutes was taken as the activation time for all subsequent trials.

**Figure 5.**
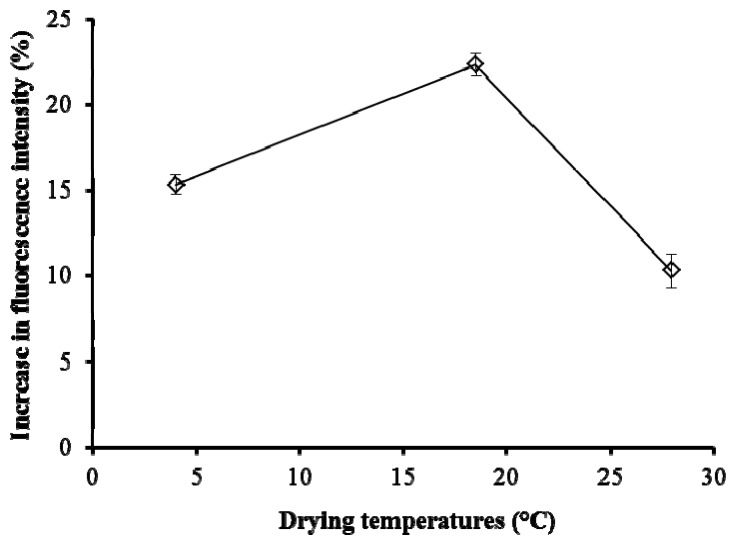
The effect of drying temperature of pHEMA to the increase of fluorescence yield.

Figure 5 shows the effect of the drying temperatures for pHEMA, with the biosensor tested on Cu 0.01 μg/L. The discs with 20 mg/mL pHEMA were dried at 4, 18.5 and 28 °C, representing the temperature of the fridge, the growth chamber, and normal room temperature, respectively. The results showed that the temperature of the growth chamber (18.5 °C) gave the highest fluorescence yield.

### 3.3. Toxicity Tests

**Figure 6.**
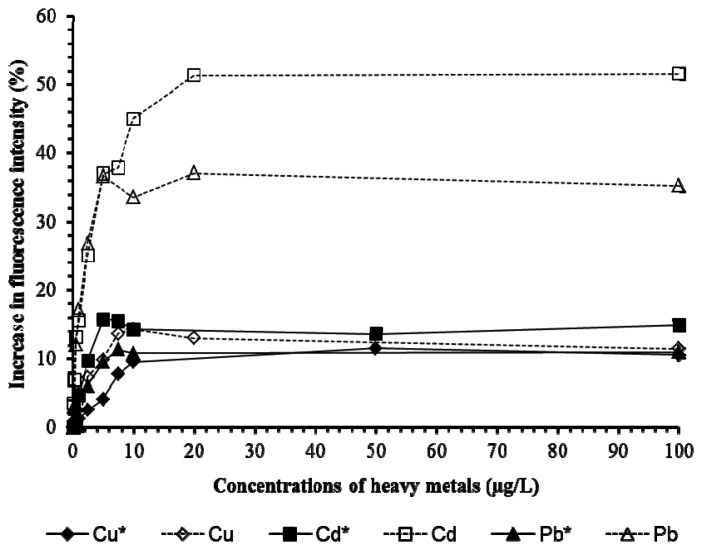
Biosensors tests on Cu, Cd, and Pb from 0–100 μg/L. The asterisk * marks the results from the tests with pHEMA.

**Figure 7.**
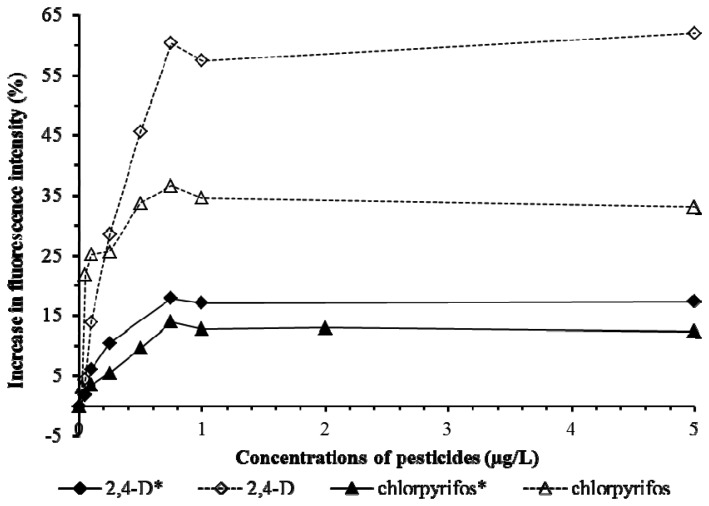
Biosensors tests on pesticides 2,4-D and chlorpyrifos. The asterisk * marks the results from the tests with pHEMA.

Tests on Cu, Cd, Pb (Figure 6) and 2,4-D and chlorpyrifos (Figure 7) showed the presence of pHEMA did not affect the trend of responses. Fluorescence yields increased proportionately to the increase of the concentration of the pollutants, and stayed at the same maximum fluorescence level at higher analyte concentrations (Figures 6 and 7). However, an overall lower fluorescence yield compared to the tests without pHEMA was evident.

Table 1 shows that the presence of pHEMA significantly affects (p < 0.05) the linear detection ranges of Pb, Cd, and chlorpyrifos, but not Cu and 2,4-D (p > 0.05). The slope values which indicate the sensitivity of the biosensor to the pollutants demonstrated that the presence of pHEMA had lower the sensitivity of the biosensor in the detection of all heavy metals and pesticides. However, the biosensor with pHEMA produced overall higher values of r^2^ (>0.9) for all the tests conducted, showing a good correlation between the fluorescence yield to the concentration of the pollutants. The experiment revealed the biosensor to be sensitive to various types of heavy metals and pesticides, which is an advantage for the screening of toxicity for a sample without identifying the source of toxicity.

### 3.4. Analytical Performance of Toxicity Biosensor

The lowest limits of detection (LLD) as described by Miller and Miller [32] for Cu, Cd, Pb, 2,4-D and chlorpyrifos with pHEMA were 1.410, 0.250, 0.500, 0.235 and 0.117 μg/L, respectively. For the biosensor without adding pHEMA, the LLD for the same pollutants were 1.195, 0.027, 0.0100, 0.025 and 0.025 μg/L respectively.

The biosensor reported in this paper showed that a change in the transduction method from electrochemical as reported by Tay *et al.* [8] to the present method using fluorescence has improved the response to various toxicants and lower the LLD by almost 1,000-fold. This is a significant achievement of this work when compared with previously reported work.

Reproducibility of the biosensor can be calculated based on RSD [33], with a value of RSD within ±20% indicating good reproducibility [34]. With pHEMA, the average RSDs of the biosensor (n = 3) for Cu, Pb, Cd, 2,4-D and chlorpyriphos were ±1.82%, ±1.35%, ±2.13%, ±1.97%, ±1.68%, respectively. The same tests without pHEMA produced RSDs of ±2.82%, ±4.80%, ±4.29%, ±3.23%, ±3.52%, respectively. The as-constructed biosensor showed very good reproducibility, with the presence of pHEMA improving the reproducibility of the biosensor by 48%.

**Figure 8.**
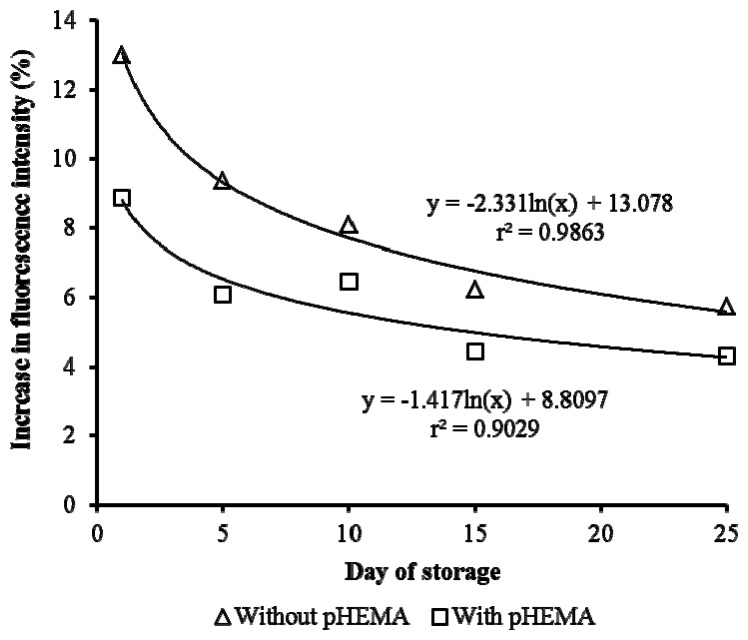
The effect of pHEMA on storability of biosensor, with the test on Cu 5 μg/L.

The storage stability of the biosensor is improved compared to those without pHEMA. This can be seen in Figure 8, where there is a sharp decrease in the response of the biosensor with storage time for cells immobilized without pHEMA. Gradual reduction of response over a 25 days' period was observed when pHEMA was used. This demonstrated the ability of the pHEMA to stabilize and maintain the cell activities. After 10 days of storage, the stability of the biosensor without pHEMA was reduced by 38.9%. The presence of pHEMA improved the stability by 11.6%. On day 25, the biosensor with pHEMA recorded fluorescence yield 5.2% better than the test without pHEMA. The decrease of fluorescence yield caused by prolonging storage was anticipated. Generally, the good correlation between storage period and fluorescence yield was indicated by the high value of r^2^ (>90%).

## 4. Conclusions

A whole cell biosensor has been developed with immobilization of cyanobacteria *A. torulosa* using pHEMA. Compared with the immobilization without pHEMA, the use of pHEMA in the biosensor was found to enhance the reproducibility, storage stability and produced better linear response correlations to the concentration of the pollutants. Although pHEMA increases the limits of detection of the biosensor, the lowest limits of detection in parts per billion still makes the biosensor with pHEMA a suitable and useful device for qualitative (especially for rapid screening) and quantitative determination of environmental pollutants such as heavy metals and pesticides. With these results, we would suggest that pHEMA is a good immobilizing polymer for filamentous cyanobacteria cells in biosensor applications.

## Figures and Tables

**Table 1.** The linear equations, the value of r^2^, linear detection ranges, and average RSD of Cu, Cd, Pb, 2,4-D and Chlorpyriphos biosensors, with asterisk * marks the tests conducted with pHEMA.

**Pollutants**	**Linear Equation**	**r^2^**	**Linear Detection Range (μg/L)**	**Average RSD (±%)**
Cu	y = 0.9957x + 5.0407	0.9402	2.50–10.00	2.49
Cu*	y = 1.3692x – 1.78	0.9531	2.50–10.00	1.63
Cd	y = 3.2958x + 14.357	0.9261	0.50–10.00	5.17
Cd*	y = 3.2259x + 0.3342	0.9507	0.50–5.00	2.95
Pb	y = 5.31x + 11.199	0.9707	0.50–5.00	5.31
Pb*	y = 1.0322x + 3.836	0.9835	1.00–7.50	2.37
2,4-D	y = 76.931x + 5.128	0.9763	0.05–0.75	3.95
2,4-D*	y = 20.702x + 3.0857	0.9206	0.05–0.75	1.50
Chlorpyrifos	y = 20.882x + 21.687	0.9526	0.05–0.75	4.48
Chlorpyrifos*	y = 16.337x + 1.5428	0.9981	0.01–0.75	2.20

## References

[1] Duruibe J.O., Ogwuegbu M.O.C., Egwurugwu J.N. (2007). Heavy metal pollution and human biotoxic effects. Int. J. Phys. Sci..

[2] Lichtfouse E., Navarrete M., Debaeke P., Souchère R., Alberola C., Menassieu J. (2009). Agronomy for sustainable agriculture: A review. Agron. Sustain. Dev..

[3] Lichtfouse E., Schwarzbauer J., Robert D. (2005). Environmental Chemistry, Green Chemistry and Pollutants in Ecosystems.

[4] Hogendoorn E., van Zoonen P. (2000). Recent and future developments of liquid chromatography in pesticide trace analysis. J. Chromatogr. A.

[5] Rogers K.R. (1995). Biosensor for environmental applications. Biosens. Bioelectron..

[6] Van der Hoff R.G., van Zoonen P. (1999). Trace analysis of pesticides by gas chromatography. J. Chromatogr. A.

[7] Védrine C., Leclerc J.C., Durrieu C., Tran-Minh C. (2003). Optical whole-cell biosensor using *Chlorella vulgaris* designed for monitoring herbicides. Biosens. Bioelectron..

[8] Tay C.C., Salmijah S., Lee Y.H. (2005). A copper toxicity biosensor using immobilized cyanobacteria. Anabaena torulosa. Sens. Lett..

[9] Frense D., Müller A., Beckmann D. (1998). Detection of environmental pollutants using optical biosensor with immobilized algae cells. Sens. Actuators B Chem..

[10] Maly J., Masojidek J., Masci A., Ilie M., Cianci E., Foglietti V., Vastarella W., Pilloton R. (2005). Direct mediatorless electron transport between the monolayer of photosystem II and poly(mercapto-p-benzoquinone) modified gold electrode-new design of biosensor for herbicide detection. Biosens. Bioelectron..

[11] Wang H., Wang X.J., Zhao J.F., Chen L. (2008). Toxicity assessment of heavy metals and organic compounds using CellSense biosensor with *E. coli*. Chin. Chem. Lett.

[12] D' Souza S.F. (2001). Review microbial biosensors. Biosens. Bioelectron..

[13] Trevan M.D., Mak L. (1988). Immobilized algae and their potential for use as biocatalysts. Trends Biotechnol..

[14] Sanders C.A., Rodriguez M., Greenbaum E. (2001). Strand-off tissue based biosensors for the detection of chemical warfare agents using photosynthetic fluorescence induction. Biosens. Bioelectron..

[15] Philp J.C., Balmand S., Hajto E., Bailey M.J., Wiles S., Whitley A.S., Lelley A.K., Hajto J., Dunbar S.A. (2003). Whole cell immobilized biosensors for toxicity assessment of a wastewater treatment plant treating phenolic-containing waste. Analy. Chim. Acta.

[16] Wong L.S., Salmijah S., Lee Y.H. (2008). Toxicity biosensor for the evaluation of cadmium toxicity based on photosynthetic behavior of cyanobacteria Anabaena torulosa. Asian J. Biochem..

[17] Brickerstaff G.F. (1997). Immobilization of Enzymes and Cells.

[18] Cantarella M., Migliaresi C., Tafuri M.G., Alfani F. (1984). Immobilization of yeast cells in hydroxyethylmethacrylate gels. Appl. Microbiol. Biotechnol..

[19] Caykara T., Özyürek C., Kantoglu Ö., Güven O. (2002). Influence of gel composition on the solubility parameter of poly(2-hydroxyethil methacrylate-itaconic acid) hydrogel. J. Polym. Sci. B.

[20] Montheard J.P., Chatzopoulos M., Chappard D. (1992). 2-hydroxyethyl methacrylate (HEMA): Chemical properties and applications in biomedical fields. J. Macromol. Sci..

[21] Ratner B.D., Hoffman A.S. (1976). Hydrogels for Medical and Related Applications.

[22] Sharina A.H., Lee Y.H., Musa A. (2009). Biosensors for phenolic compounds by immobilization of tyrosinase in photocurable methacrylic-acrylic membranes of varying hydrophilicities. Anal. Sci..

[23] Norazlina O., Fatimah A.B., Abu B.S., Lee Y.H., Rahman W. (2006). A preliminary investigation on a histamine biosensor constructed from diamine oxidase immobilized onto a oxygen probe. Malaysia J. Anal. Sci..

[24] Tan L.L., Musa A., Lee Y.H. (2011). Determination of ammonium ion using a reagentless amperometric biosensor based on immobilized alanine dehydrogenase. Sensors.

[25] Krause G.H., Weis E. (1991). Chlorophyll fluorescence and photosythesis: The basic. Annu. Rev. Plant Physiol..

[26] James D.E. (1978). Culturing Algae.

[27] Trainor F.R. (1979). Introductory Phycology.

[28] Carr N.G., Whitton B.A. (1982). The Biology of Cyanobacteria.

[29] Gil C.G., Kim Y.J., Gu M.B. (2002). Enhancement in the sensitivity of a gas biosensor by using an advanced immobilization of a recombinant bioluminescent bacterium. Biosens. Bioelectron..

[30] Papageorgiou G.C. (2004). Chlorophyll A Fluorescence in Advances in Photosynthesis and Respiration.

[31] Chouteau C., Dzyadevych S., Durrieu C., Chovelon J.M. (2005). A bi-enzymatic whole cell conductometric biosensor for heavy metal ions and pesticides detection in water sample. Biosens. Bioelectron..

[32] Miller J.N., Miller J.C. (2000). Statistic and Chemometrics for Analytical Chemistry.

[33] Zheng H., Xue H., Zhang Y., Shen Z. (2002). A glucose biosensor based on microporous polyacrylonitrile synthesized by single rare-earth catalyst. Biosens. Bioelectron..

[34] Razola S.S., Pochet S., Grosfils K., Kauffmann J.M. (2003). Amperometric determination of choline released from rat submandibular gland acinar cells using a choline oxidase biosensor. Biosens. Bioelectron..

